# Derivation of High Purity Neuronal Progenitors from Human Embryonic Stem Cells

**DOI:** 10.1371/journal.pone.0020692

**Published:** 2011-06-06

**Authors:** Gabriel Nistor, Monica M. Siegenthaler, Stephane N. Poirier, Sharyn Rossi, Aleksandra J. Poole, Maura E. Charlton, John D. McNeish, Chris N. Airriess, Hans S. Keirstead

**Affiliations:** 1 Reeve-Irvine Research Center, Sue and Bill Gross Stem Cell Research Center, Department of Anatomy and Neurobiology, School of Medicine, Sue & Bill Gross Hall, a CIRM Institute, University of California Irvine, Irvine, California, United States of America; 2 California Stem Cell, Inc., Irvine, California, United States of America; 3 Pfizer Regenerative Medicine, Cambridge, Massachusetts, United States of America; Baylor College of Medicine, United States of America

## Abstract

The availability of human neuronal progenitors (hNPs) in high purity would greatly facilitate neuronal drug discovery and developmental studies, as well as cell replacement strategies for neurodegenerative diseases and conditions, such as spinal cord injury, stroke, Parkinson's disease, Alzheimer's disease, and Huntington's disease. Here we describe for the first time a method for producing hNPs in large quantity and high purity from human embryonic stem cells (hESCs) in feeder-free conditions, without the use of exogenous noggin, sonic hedgehog or analogs, rendering the process clinically compliant. The resulting population displays characteristic neuronal-specific markers. When allowed to spontaneously differentiate into neuronal subtypes *in vitro*, cholinergic, serotonergic, dopaminergic and/or noradrenergic, and medium spiny striatal neurons were observed. When transplanted into the injured spinal cord the hNPs survived, integrated into host tissue, and matured into a variety of neuronal subtypes. Our method of deriving neuronal progenitors from hESCs renders the process amenable to therapeutic and commercial use.

## Introduction

Central to the use of pre-differentiated cells for screening or transplantation is the purity of the cell population. Cell sorting requires that the desired cells be labeled with fluorescent antibodies to phenotype-specific cell surface proteins, or engineered to express a fluorescent protein under the control of a phenotype-specific promoter. Both techniques have potential drawbacks: immunodetection necessitates further digestion of cell surface proteins to remove the antibody label, and reporter gene expression requires genetic manipulation of the starting population. Both of these sorting methods require time, which allows for amplification of contaminant populations. Regardless of the technique, the purity of the sorted population is dependent on the specificity of the label or reporter used. In the event that a cell-specific promoter is available, a conjunctional method is the knock-in of a gene for a foreign protein such as green fluorescent protein under the control of the promoter to highlight the cells for selection. However, the tumorigenic risk posed by insertion methods renders the use of reporter genes or any cell-specific selection genes, such as antibiotic resistance genes, undesirable for clinical use as per current US FDA guidelines. Lastly, the small yield of sorting methods limits the commercial or clinical viability of the approach. Nonetheless, these sorting methods are very useful for the production of small scale research grade cell populations.

Several groups have described the derivation of neural progenitors from hESCs, which are capable of differentiating into neuronal and glial cell types; indeed, neural induction appears to be the default path from hESC [1], [2], [3], [4]. Neuronal progenitors are more lineage-specific than neural progenitors, as they do not differentiate into oligodendrocytes and astrocytes. Human neurons and neuronal progenitors are difficult to obtain from primary culture of harvested tissue, and impossible to obtain in large quantities for clinical and commercial application. As there is growing application of human stem cell technologies in the biopharmaceutical sector, the availability of human neuronal progenitors (hNPs) in high purity would greatly facilitate neuronal drug discovery and lead developmental studies. In addition, cell replacement strategies for neurodegenerative diseases and conditions will be facilitated with reproducibly manufactured, high purity hNPs.

Here we describe for the first time a method for producing hNPs in large quantity and high purity from hESCs in serum-free and feeder-free conditions, without the use exogenous noggin or sonic hedgehog. With appropriate quality and manufacturing control, this method could produce clinical grade hNPs in sufficient quantity for use in biopharmaceutical research and developmental studies, as well as therapeutic strategies addressing diseases and conditions characterized by the loss of neurons.

## Materials and Methods

### Ethics Statement

All animal work for this study was approved (approval ID number 2007–2725) and carried out in accordance with the UCI Institutional Animal Care and Use Committee. Animals received appropriate post-surgical care including subcutaneous saline, prophylactic Baytril (2.5 mg/kg/d, s.c.; Bayer, Shawnee Mission, KS), and Buprenorphine (0.025 mg/kg/d, s.c.; Western Medical Supply, Los Angeles, CA) for three days. Animals were inspected for weight loss, dehydration, discomfort, and autophagia, with appropriate veterinary care as needed. All work involving human embryonic stem cells was approved by the UCI Human Embryonic Stem Cell Research and Oversight Committee (2007–5645).

### Differentiation of hNPs from hESCs

hNPs were derived from hESC lines H7, hCSC14 and hCSC14-CL1 (California Stem Cell, Inc., Irvine, CA) at passages 15–17. hESC cultures were expanded on Matrigel (BD Biosciences, San Jose, CA) or a defined substrate, CellGel (California Stem Cell, Inc., Irvine, CA). StemBlast (California Stem Cell, Inc., Irvine, CA) was used to feed the cultures daily and was supplemented with 10 ng bFGF/ml/day as previously described [5]. When cultures attained >75% confluence, cells were removed from the adherent substrate, transferred to ultra low binding 75 cm^2^ or 225 cm^2^ or 630 cm^2^ dishes (Corning, NY) and suspended in NeuroBlast media (California Stem Cell, Inc., Irvine, CA), a DMEM-F12 based media absent of bone morphogenic proteins and pluripotenfig.cy sustaining factors, that induces ectodermal commitment. NeuroBlast media was modified by addition of Glutamax (Invitrogen, Carlsbad, CA) diluted to 1× from stock, and B27 supplement (Invitrogen, Carlsbad, CA) diluted to 1× from stock. FGF (Millipore, Billerica, MA) and retinoic acid (all-trans-retinoic acid; RA; Sigma Aldrich, St. Louis, MO) was added to the cultures at a final concentration of 10 µM in DMSO daily for 5 days. After the RA treatment, the cultures were fed every second day, and FGF was reduced from 10 ng/ml to 5 ng/ml. The feeding procedure consisted of a 3–5 minute gravity selection of the dense cell clusters in a column (50 ml centrifuge tube) followed by complete replacement of the supernatant, which contained the cell clusters of lesser density. At day 18, the cultures were plated on Matrigel or CellGel and left to spontaneously differentiate for two days. At day 20, the cells were dissociated again. Cells were then either 1) replated into flasks and maintained for up to 3 weeks with no addition of growth factors for polymerase chain reaction (PCR) analysis of gene expression, 2) were plated into 96-well plates at a density of 10,000 and 40,000 cells per well and maintained for 3 weeks by 2/3 media replacement every 2^nd^ day in order to assess the spontaneous differentiation into neuronal subtypes via immunocytochemistry, or 3) were plated into 96-well plates with manipulated culture conditions and maintained for 3 weeks in order to assess the altered differentiation into neuronal subtypes via immunocytochemistry. During the 3 week period of spontaneous differentiation, no growth factors were added to the cultures. For the 3 week period of manipulated differentiation, NeuroBlast media was modified with RA free B27 supplement and the addition of either FGF8 (10 ng/ml), FGF2 (10 ng/ml), BDNF (10 ng/ml), GDNF (10 ng/ml), or Activin A (5 ng/ml).

### Immunocytochemical Labeling

For hNP identification and neuronal subtyping, cultures were fixed with 4% paraformaldehyde for one hour, blocked with 5% serum, and then exposed to primary antibodies anti-nestin 1∶200, anti-TUJ1 1∶400, anti-ChAT 1∶200, anti-O4 1∶200, anti-MAP2 1∶200, anti-Musashi-1 1∶200, anti-GABA_A_ Receptor α1 1∶200 (Chemicon, Temecula, CA/Millipore, Billerica, MA), anti-GFAP 1∶500 (Sigma), anti MNR2 (HB9) 1: 100 (Developmental Studies Hybridoma Bank, Iowa City, IA), anti-Tau 1∶200, anti-DARPP32 1∶200 (Cell Signaling Technology, Danvers, MA), anti-Ki67 1∶1000 (DAKO, Carpinteria, CA), anti-doublecortin 1∶200, anti-PDGF-a 1∶200, anti-NG2 1∶200 (Santa Cruz Biotechnology, Santa Cruz, CA), anti-tyrosine hydroxylase 1∶200, anti-5HT 1∶200 (Abcam, Cambridge, MA) at 4°C overnight. Primary antibody application was followed by fluorescent secondary antibodies (Alexafluor-594 or Alexafluor-488 conjugated; Invitrogen, Carlsbad, CA), and visualized with fluorescence microscopy according to standard protocols.

Dividing cells were labeled using a BrdU labeling and detection kit (Roche, Mannheim, Germany). Briefly, adherent cells at 50% confluency were incubated with BrdU labeling medium for 1 hour at 37°C, then washed 3 times prior to fixation. Mouse anti-BrdU working solution was then applied to fixed cells and incubated for 30 minutes at 37°C. After washing 3 times, cells were incubated with mouse anti-IgG solution for 30 minutes at 37°C. Cells were then washed, mounted, and visualized with fluorescent microscopy.

### Real-Time Polymerase Chain Reaction

RNA from hNPs that were allowed to spontaneously differentiate was harvested every seven days for three weeks after the hNP differentiation protocol (Days 7, 14, and 21 days after hNP differentiation). Cells were collected after trypsinization with 0.25% trypsin EDTA and total RNA was extracted using the RNeasy kit and RNase-free DNase set (Qiagen, Valencia, CA) according to the manufacturer's instructions. RNA concentrations were measured with the NanoDrop ND-8000 Spectrophotometer (NanoDrop Technologies, Wilmington, DE). One microgram (1 µg) of total RNA was reverse transcribed using RT^2^ First Strand Kit (SABiosciences, Frederick, MD) according to the manufacturer's instructions. This kit contains a procedure to eliminate contaminating genomic DNA from RNA samples before reverse transcription. cDNA was then assayed on a human neurogenesis and neural stem cell RT^2^ profiler PCR array (SABiosciences, Frederick, MD) which profiles 84 genes related to the processes of neurogenesis and neural stem cell differentiation. Real-time polymerase chain reaction (PCR) measurements were performed on an ABI PRISM 7900HT Sequence Detector System (Applied BioSystems, Foster City, CA, http://www.appliedbiosystems.com).

Gene expression that changed by 8-fold, as determined by the ΔΔCt method is reported.

### Spinal Cord Injury and Transplantation

All animal work was carried out in accordance with the UCI Institutional Animal Care and Use Committee (2007–2725). Adult, female Sprague-Dawley rats (Charles River Laboratories; San Diego, CA) underwent a laminectomy at C5–C6 and received a 200 kD bilateral contusion injury using an Infinite Horizons Impactor (Precision Systems and Instruments; Lexington, KY). Seven days later, the laminectomy site was re-exposed, the animals were secured to a stereotactic apparatus, and 100,000 hNP cells in total were transplanted into the spinal cord. Cells were injected bilaterally cranial and caudal to the injury site using a 33G needle attached to a Hamilton syringe at 25,000 cells/uL. One day prior to transplant, the animals received cyclosporine A (20 mg/kg/d, s.c.; Bedford Laboratories, Bedford, OH), which was then continued for the duration of the protocol. In addition, animals received 0.5 mg/kg Rolipram (A.G. scientific; San Diego, CA) subcutaneously from 2 days prior to transplant and for thirty days thereafter.

hNP cells were dissociated from adherent conditions the night prior to transplantation. The cells were then plated into a non-adherent flask to form microspheres. The cells were collected and spun at 100rcf. The pellet was resuspended in Cell Transplant Solution (CTS; California Stem Cell, Inc., Irvine, CA) and 2 more centrifugations and resuspensions were performed to wash the hNP microspheres. This method was performed to remove non-viable cells.

### Histology

All animals were sacrificed 3 months post-transplant via trans-cardiac perfusion with 4% paraformaldehyde. The tissue was cryopreserved in 27% sucrose and the cords were sectioned into 1 mm blocks and embedded in OCT compound. Tissue was cryosectioned at 20 um and mounted onto gelatin coated slides. To determine the fate of the transplanted hNP cells, the tissue was labeled for human nuclei 1∶200 (Abcam, Cambridge, MA), TUJ1 1∶1000 (Covance, Princeton, NJ), doublecortin 1∶200 (Santa Cruz Biotechnology, Santa Cruz, CA), ChAT 1∶100 (Millipore, Temecula, CA), GAD 65–67 1∶500 (Abcam, Cambridge, MA), anti-p75 neurotrophin receptor 1∶200 (Santa Cruz Biotechnology, Santa Cruz, CA), 5-HT 1∶200 (Abcam, Cambridge, MA), NCAM 1∶500 (Santa Cruz Biotechnology, Santa Cruz, CA) and synaptophysin 1∶200 (Abcam, Cambridge, MA). Secondary AlexaFluor antibodies (Invitrogen, Carlsbad, CA) against primary antibody species were used for fluorescent imaging, and biotinylated secondary antibodies were used with DAB reaction for light microscopy imaging.

## Results

### In Vitro Differentiation Profile

**Figure 1:**
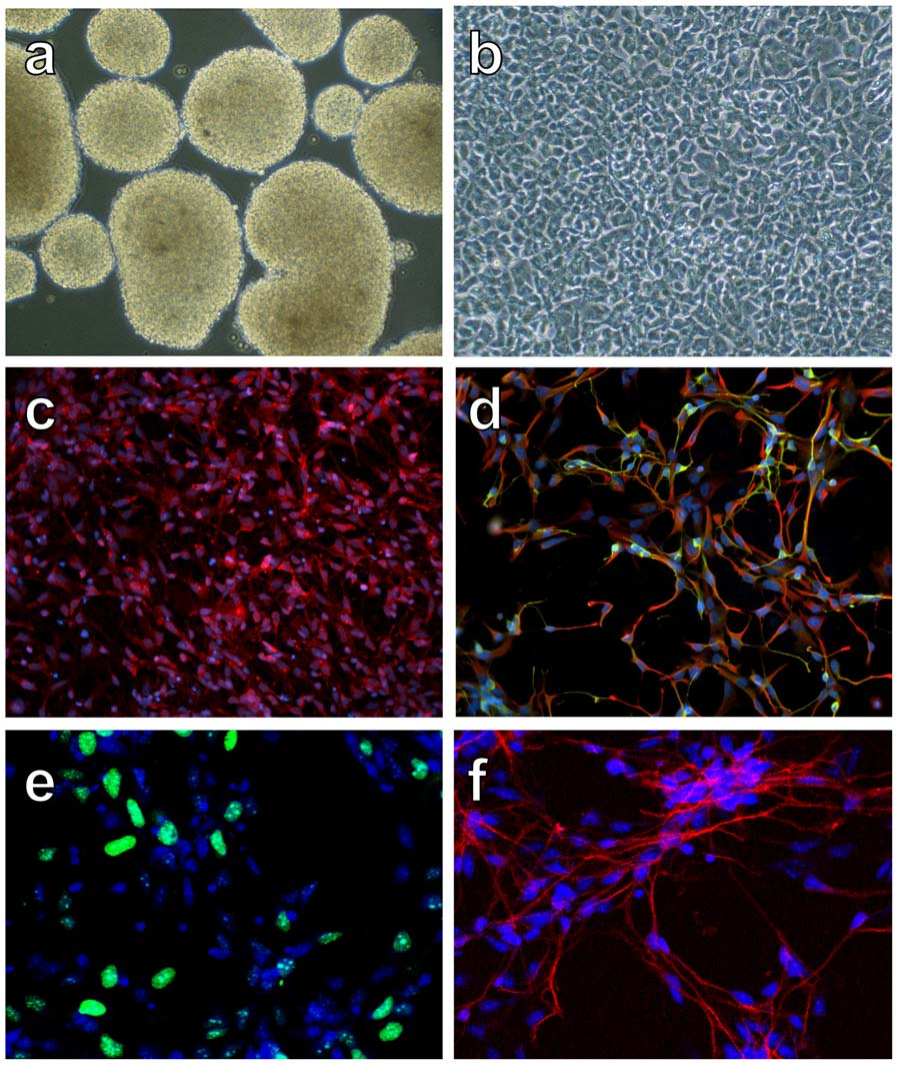
Differentiation of hESCs into neuronal progenitor cells. A) Neurospheres at day 14 of differentiation. B) After plating of neurospheres, neuroepithelial cells displayed a typical morphology by days 16–18 of differentiation. C) Musashi-1immunolabeling of cells. D) Nestin (red) and TUJ1 (green) immunolabeling of cells demonstrated a high percentage of co-localization. E) Ki67 immunolabeling revealed that a portion of hNPs are mitotic. F) Doublecortin immunolabeling revealed a high percentage of new neurons.

At day 14 of the 21-day differentiation protocol, free-floating cultures consisted of solid core neurospheres (Fig. 1a). Following plating of neurospheres, neuroepithelial cells were identified by their characteristic morphology at days 16–18 of differentiation (Fig. 1b). Immunocytochemical staining revealed extensive expression of the RNA binding protein, Musashi-1, which was observed in 99.2% of cells (Fig. 1c), and nestin (red) in 98.8% of cells (Fig. 1d), indicating that the culture consisted of high purity neural progenitor cells. Co-expression of TUJ1 (green) in some of the nestin (red) positive cells, and the presence of typical neurites on all cells, further suggested the differentiation of the cultures towards neuronal types (Fig. 1d). No cells labeled with NG2 or PDGF-a, indicating that cultures did not contain cells of the oligodendroglial lineage.

As gross observation of cultures suggested continuous expansion of the cultures, we investigated the proliferative potential of the hNPs. Our culture and passage conditions did not group dividing cells in neural rosettes as is common for other culture conditions, but rather, proliferating cells were homogenously dispersed. The proliferation marker Ki67 was observed in 26.4% of cells (Fig. 1e) and BrdU labeling was observed in 28.6% of cells (data not shown). Doublecortin, the microtubule-associated protein that is expressed by the neuronal lineage, was detected in 91.2% of the cells (Fig. 1f), further supporting the interpretation that our cultures consisted of high purity neuronal progenitors. Consistent with our Ki67 and BrdU data, the amount of RNA extracted from cultures was increased by 28.4% after 7 days of spontaneous differentiation in growth factor free media (after day 21 of neuronal progenitor differentiation), indicative of cell proliferation.

**Figure 2:**
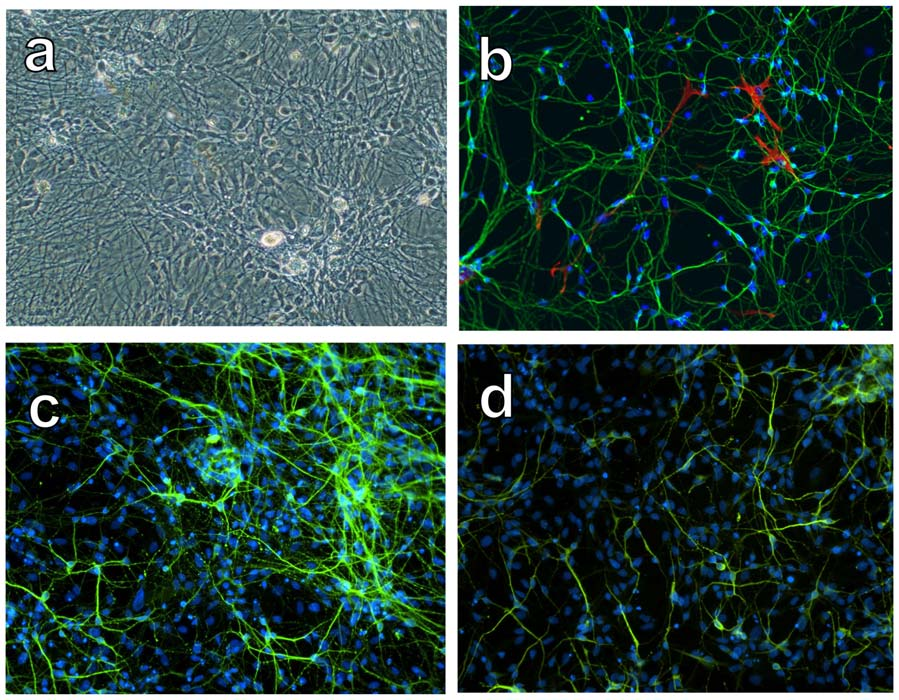
Differentiation of hNPs into neurons after 3 weeks of maturation. A) hNP-derivates displayed a highly branched morphology, consistent with a neuronal phenotype. B) Immunolabeling with TUJ1 (green) and GFAP (red) revealed a high purity neuronal culture with few astrocytes. C) Cells were immunopositive for Tau, a microtubule associated protein, consistent with neuronal differentiation. D) Cells were immunopositive for MAP2, a microtubule associated protein, consistent with neuronal differentiation.

**Figure 3:**
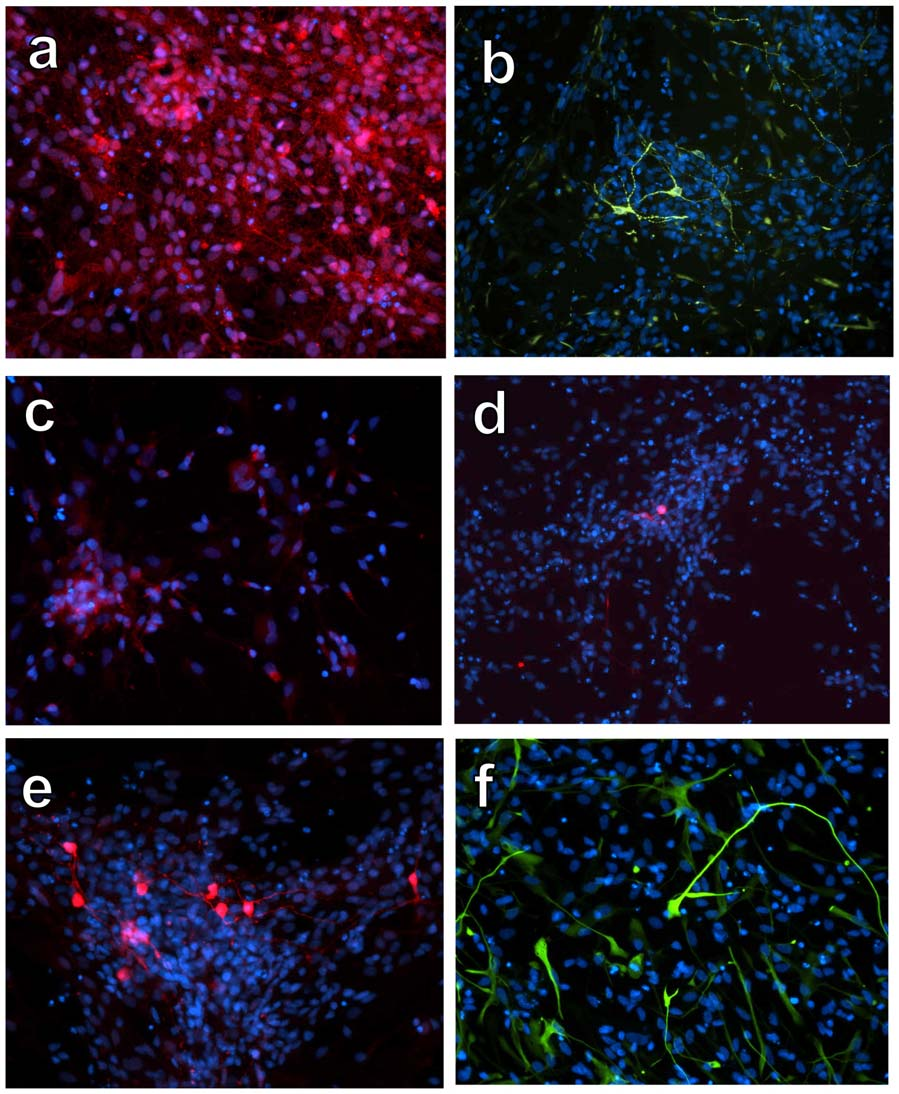
Differentiation of hNPs into neuronal subtypes after 3 weeks of maturation. A) Cells were immunopositive for ChAT, demonstrating that the majority of hNPs have the potential to become cholinergic neurons. B) 5HT expression was detected in a subset of cells, demonstrating that hNPs have the potential to become serotonergic neurons. C) GABA_A_ receptor α1 expression was detected in a subset of cells, demonstrating the ability to become GABA-responsive neurons. D) TH expression was detected in a subset of cells, demonstrating the ability to become dopaminergic neurons. E) DARPP32 expression was detected in a subset of cells, demonstrating the ability to become striatal interneurons. F) Few GFAP positive cells were identified in matured cultures.

The spontaneous differentiation potential of this cell population was assessed by immunocytochemical and RNA expression analyses after 3 weeks in growth factor free conditions. hNP-derivates displayed a highly branched morphology (Fig. 2a). Immunocytochemical staining confirmed neuronal maturation; 96.2% of cells expressed TUJ1 (green; Fig. 2b), 95.4% of cells expressed the microtubule-associated protein Tau (Fig. 2c), and 80.5% of cells expressed the neuron-specific microtubule associated protein MAP2 (Fig. 2d). 4.1% of cells expressed GFAP (red; Fig. 2b). Choline acetyltransferase (ChAT) expression was detected in 97% of cells, demonstrating that the majority of hESC-derived hNPs have the potential to become cholinergic neurons (Fig. 3a). GABA_A_ receptor α1 expression was detected in 87% of hNP-derivates, demonstrating the capability to become GABA-responsive neurons (Fig. 3c). hNP-derivates also demonstrated the ability to become other neuronal subtypes, as evidenced by their expression of 5-HT (3%; Fig. 3b), tyrosine hydroxylase (1%; Fig. 3d), and DARPP32 (2%; Fig. 3e). A small percentage of cells (4.1%) expressed the astrocyte marker GFAP (Fig. 2b, 3f). No cells were labeled with 04, indicating that cultures did not contain cells of the oligodendroglial lineage. <0.1% of cells were unlabeled. Based on morphology, size, ChAT and GABA_A_ receptor α1 expression expression, we conclude that most of the cells that resulted from spontaneous differentiation of high purity NPs were motor neuron progenitors.

**Figure 4:**
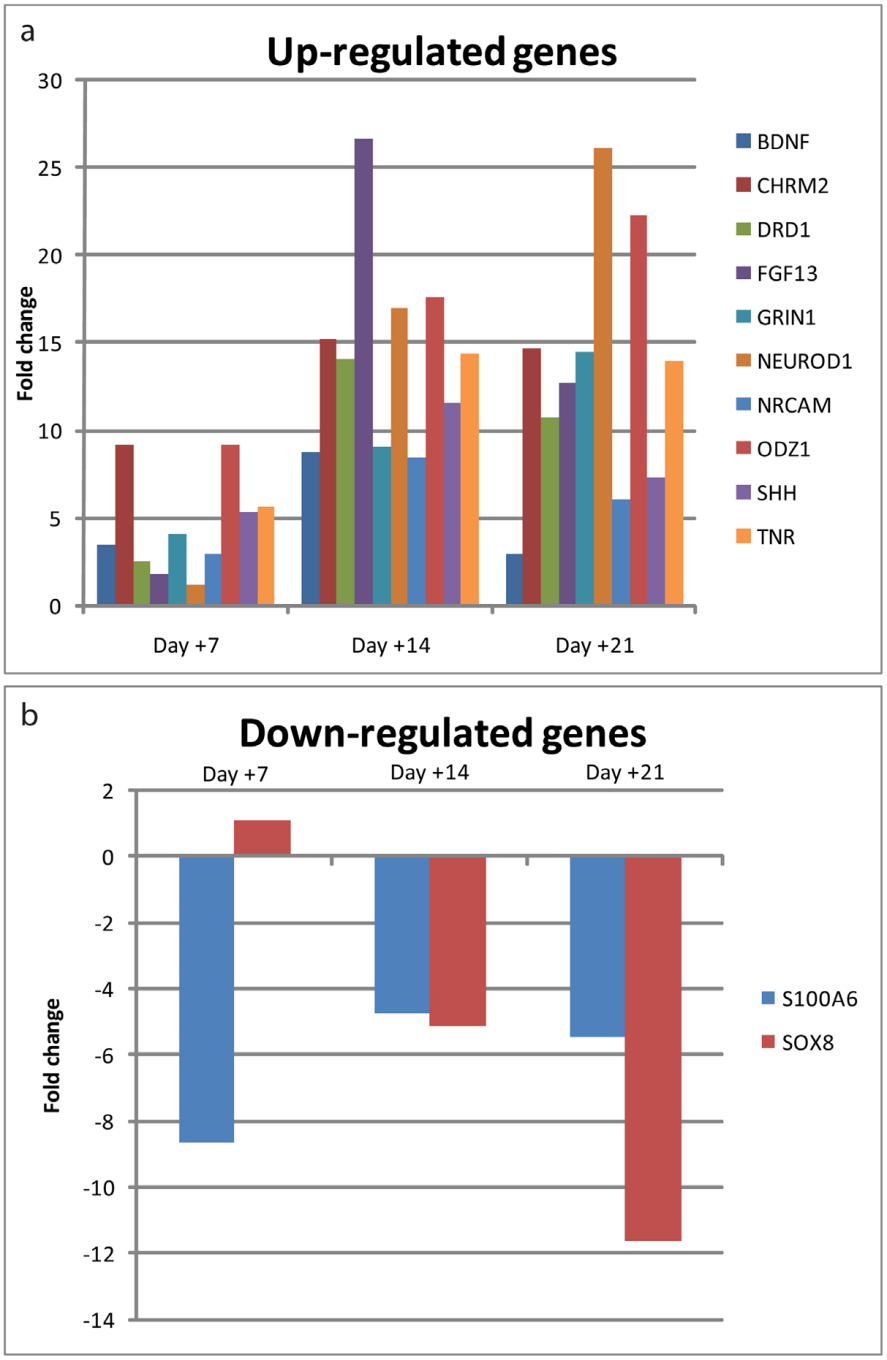
RNA expression of matured cultures is consistent with a neuronal lineage. A) Several neurogenic genes were up-regulated after 3 weeks of maturation, as compared to undifferentiated hNPs. B) S100A6 and SOX8 genes, which are often expressed in glial cells, were down-regulated in the matured cultures, as compared to undifferentiated hNPs.

In these growth factor free conditions, RNA analyses indicated that hNP-derivates up-regulated expression of brain-derived neurotrophic factor (BDNF), neuronal cell adhesion molecule (NCAM), and sonic hedgehog (SHH) by 8-fold as compared to undifferentiated hNPs (after the 21 day differentiation protocol; Fig. 4a). The muscarinic cholinergic receptor 2 (CHRM2), dopamine receptor D1 (DRD1), fibroblast growth factor 13 (FGF13), ionotropic N-methyl D-aspartate glutamate receptor 1 (GRIN1), neurogenic differentiation 1 (NEUROD1), and odz, odd Oz/ten-m homolog 1 (ODZ1) were up-regulated by 10-fold as compared to undifferentiated hNPs (after the 21 day differentiation protocol; Fig. 4a). This RNA expression profile is consistent with neuronal differentiation.

In these growth factor free conditions, RNA analyses indicated that hNP-derivates down-regulated expression of the developmentally regulated S100 Ca(2+)-binding protein family member S100A6 and the oligodendrocyte marker SOX8 by 8-fold as compared to undifferentiated hNPs (after the 21 day differentiation protocol; Fig. 4b). This RNA expression profile is consistent with developmental maturation of the neuronal lineage.

### In Vivo Differentiation Profile

**Figure 5:**
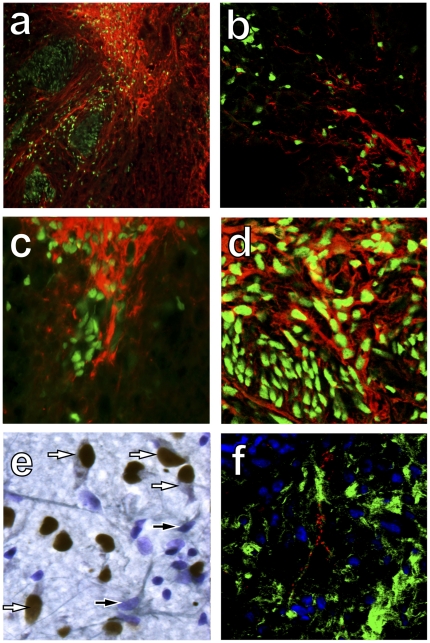
Differentiation of hNPs 3 months after transplantation to spinal cord injury sites. A) Human nuclei-positive cells (green) expressed TUJ1 (red), consistent with a neuronal phenotype. B) Human nuclei-positive cells (green) expressed doublecortin (red), consistent with a young neuronal phenotype. C) Human nuclei-positive cells (green) expressed p75 (red), consistent with young motor, sensory and sympathetic neurons. D) Human nuclei-positive cells (green) expressed GAD 65–67 (red), consistent with an interneuronal phenotype. E) Human nuclei-positive cells (brown) expressed ChAT (gray), consistent with a cholinergic phenotype (nuclear counterstain in purple). F) Human specific NCAM (green) and synaptophysin (red) immunolabeling suggests integration of transplanted cells with the host environment.

The differentiation potential of hNPs was further analyzed by assessing their phenotype 3 months following transplantation into sites of spinal cord injury. Immunohistochemical analyses indicated that anti-human nuclei positive cells co-localized with the neuronal specific markers TUJ1 (Fig. 5a) and doublecortin (Fig. 5b). Neuronal subtype specific differentiation was evidenced by double labeling of anti-human nuclei positive cells with p75 consistent with their differentiation to young motor, sensory or sympathetic neurons (Fig. 5c), GAD 65–67 consistent with their differentiation to interneurons (Fig. 5d), and ChAT consistent with their differentiation to cholinergic neurons (Fig. 5e). NCAM (green) and synaptophysin (red) double immunolabeling revealed synapses on anti-human nuclei positive cells (Fig. 5f). This immunohistochemical profile indicates that hNPs have the capability of differentiating into neuronal subtypes, a process that is likely controlled by the environmental factors to which they are exposed.

### Manipulation of In Vitro Differentiation Profile

**Figure 6:**
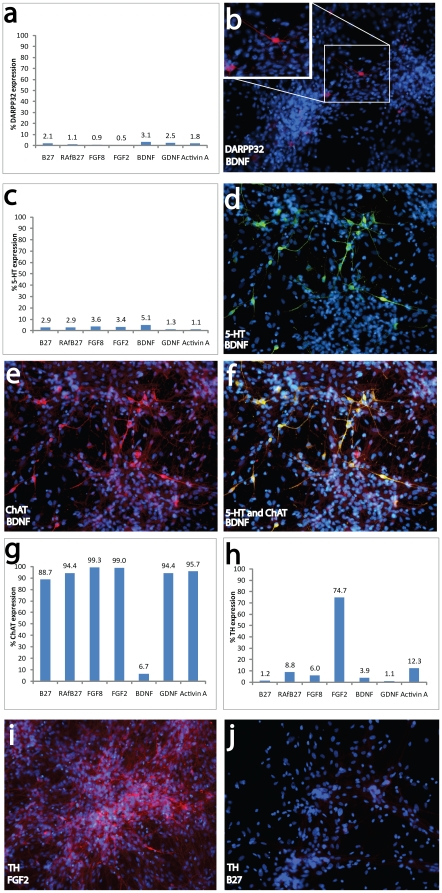
Culture manipulation altered the percentage of neuronal subtype derivates. In RA free conditions with the addition of factors (A), DARPP32 expression was detected in a subset of cells (B), demonstrating the ability to become GABA-responsive neurons. In RA free conditions with the addition of factors (C), 5-HT expression was detected in a subset of cells (D), demonstrating the ability to become serotonergic neurons. In RA free conditions with the addition of BDNF, ChAT expression was detected in a subset of cells (E), demonstrating the ability to become cholinergic neurons. F) In RA free conditions with the addition of BDNF, 5-HT and ChAT colocalize. G) Alteration of growth factors in RA-free conditions changes ChAT expression. In RA free conditions with the addition of FGF2 (H), TH expression was detected in a subset of cells (I), demonstrating the ability to become dopaminergic neurons. J) In RA-containing conditions, few or no TH positive cells were present.

The ability of hNPs to differentiate into a variety of neuronal subtypes in vivo suggested that their differentiation profile could be manipulated in vitro. The differentiation potential of hNPs was assessed 3 weeks following various manipulations of culture conditions. RA–free conditions resulted in a decrease of DARPP32 expression (B27 and RAfB27 in Fig. 6a; 2.1% and 1.1% respectively). However, the addition of BDNF or GDNF to RA-free conditions resulted in an increased number of cells expressing DARPP32 above that seen in control conditions (BDNF and GDNF in Fig. 6a; 3.1% and 2.5% respectively). The addition of Activin A to RA-free conditions yielded a similar percentage of cells expressing DARPP32 to that of RA-containing condition (Activin A in Fig. 6a; 1.8%). The addition of FGF8 and FGF2 to RA-free conditions decreased the DARPP32 intensity to levels below that of RA-free conditions (FGF8 and FGF2 in Fig. 6a; 0.9% and 0.5% respectively).These data demonstrate that hNPs can differentiate into DARPP32 expressing neurons, consistent with a medium spiny neuronal phenotype.

Although RA-free conditions (RAfB27 in Fig. 6c) resulted in a percentage of 5-HT expressing cells that was not different than RA-containing conditions (B27 in Fig. 6c), the addition of FGF8, FGF2 and BDNF to RA-free conditions led to an increase in the percentage of 5-HT expressing cells (Fig. 6d). On the other hand, the addition of GDNF and Activin A to RA-free conditions resulted in a decrease of 5-HT expressing cells (1.3% and 1.1% respectively). Double-labeling studies revealed that several 5-HT positive cells were also ChAT positive (Fig. 6e, f). The addition of FGF8 and FGF2 to RA-free conditions increased the percentage of ChAT expressing cells (Fig. 6g), while the addition of BDNF decreased it. These data demonstrate that hNPs can differentiate into 5-HT expressing neurons, similar to those found within the raphe dorsalis.

While most conditions resulted in a similar percentage of tyrosine hydroxylase positive (TH) cells, the addition of FGF2 to RA-free conditions increased the percentage of TH expressing cells (Fig. 6i, j). These data demonstrate that hNPs can differentiate into TH expressing neurons, similar to those found within the nigrostriatal pathway.

## Discussion

The promise of hESCs resides in their ability to produce high purity lineage-specific cell populations that are difficult or impossible to obtain from primary culture of harvested tissue. Included in this category are neuronal progenitors, cells of the central nervous system (CNS) that are sensitive to mechanical and chemical injuries, do not regenerate following injury, and can not be recovered from harvested tissue in high purity. Although several studies document the directed differentiation of neural progenitors from hESCs, the glial progeny of this population limits its usefulness in neuronal developmental studies or screening assays. Here we describe a novel method for producing neuronal progenitors in large quantity and high purity from hESCs in feeder-free conditions, without the use of exogenous noggin, Shh or analogs, rendering the process clinically compliant.

Published protocols for the in vitro differentiation of embryonic stem cells into neuronal populations have invariably used Shh, bone-morphogenic protein (BMPs), noggin and Wnts alone or in combinations, and most of the authors agree that RA in combination with Shh is a reliable means of differentiating neuronal progenitors from hESCs, albeit in low purity and limited yield [6], [7], [8], [9].

The differentiation protocol described here is fundamentally different from other published protocols in the media composition and the physical manipulations of the cells during culture. With regards to media composition, this protocol differs from others in the concentration and duration of RA exposure, FGF administration, and the absence of exogenous Shh. The addition of 5 ng/ml FGF during the entire differentiation protocol is essential for high purity hNP cultures; addition of a higher dose of FGF than used here resulted in delayed differentiation and increased contamination with astrocytes and/or neural progenitors. The absence of FGF, or limited exposure to FGF, resulted in maturation of cells inside of the spheres, leading to dissociation difficulties after plating, and gross culture heterogeneity. Finally, the absence of exogenous Shh was a unique feature of this neuronal progenitor differentiation protocol.

With regards to physical manipulations, the use of non-adherent conditions at early stages of the differentiation protocol was critical for the generation of high purity cultures. Others have described culture on an adherent substrate resulting in the formation of neural rosettes (Li et al., 2005), and differentiation of low purity (10–30%) hNPs with contaminants including neural progenitors, mesodermal lineages, fibroblasts and myoblasts. To further enhance the purity of the hNP culture, our protocol used daily gravity feeding during the initial phases of differentiation to select a population which spontaneously forms neurospheres, and eliminate single cells which generate non-neuronal contaminant populations. We attribute the “spherogenic” properties to NCAM homophilic interactions on the neuronal progenitors' cell surface, with agglomeration of the cells resulting in higher density particles with faster sedimentation. Quantitative PCR analyses indicated that NCAM was significantly increased at day 14 of the differentiation protocol (data not shown). We further hypothesize that neurospheres at this stage of differentiation (12–14 days) secrete and trap enough endogenous Shh to induce hNP differentiation without the addition of exogenous Shh. Differentiation of the same hESC cultures without dissociation and feeding every 2–3 days during the first two weeks resulted in a heterogeneous population of mixed neurons, glial cells, and undifferentiated cells.

The absence of exogenous Shh enhances the clinical compliance of our hNP protocol, given the association of exogenous Shh with tumor formation [10], [11], [12], [13] and cancer cell motility/invasiveness [14]. As an alternative to the addition of exogenous Shh, our protocol involved attenuation of a developmentally important opposing signal to Shh, BMPs. Thus, clearance of BMPs and/or cells producing BMPs from the cultures would have an effect similar to the addition of Shh and/or noggin. BMP levels were attenuated in our culture conditions by elimination of serum and serum replacement. Serum contains BMPs and dorsal signaling molecules, and use of serum in culture conditions promotes the generation of dorsal phenotypes [15].

We also avoided the use of leukemia inhibitory factor (LIF) in our differentiation protocol to enhance homogeneity of the culture, as previous findings suggest that LIF and LIF receptors are implicated in astrocyte differentiation in vivo and in vitro through STAT3 [16], [17], [18]. In combination with RA, LIF acts through an epigenetic mechanism to activate the GFAP promotor [18]. Although LIF is typically not used in human cultures as it is used in mouse embryonic stem cell cultures, it is possible that embryonic feeders could secrete various factors, including LIF, or that the presence of other cell types could directly induce astrocytic differentiation. In co-cultures of undifferentiated stem cells with endothelial cells or media previously exposed to endothelial cultures, neuronal differentiation was inhibited [19], likely due to secretion of LIF [20]. Thus the presence of LIF in other attempts to derive neuronal cell types likely predisposes those populations towards glial phenotypes when exposed to RA.

hNPs expressed Musashi-1, nestin, TUJ1 and doublecortin, markers consistent with a neuronal progenitor phenotype. Mushashi-1 is a member of the highly conserved Musashi family of RNA-binding proteins, and is expressed at high levels in undifferentiated neuronal progenitor cells [21], [22], [23]. Although Musashi-1and nestin are also expressed in neural progenitor cells, which have the potential to differentiate into both neuronal and glial cell types, expression of TUJ1 and doublecortin by hNPs demonstrates their neuronal progenitor phenotype. Immunocytochemical staining with the oligodendroglial lineage marker O4 and the astrocyte marker GFAP indicated limited expression in end-stage cultures, consistent with a high purity neuronal progenitor population. Furthermore, when allowed to spontaneously differentiate, hNPs generated cholinergic, serotonergic, dopaminergic and/or noradrenergic, and medium spiny striatal neuronal sub-types. Manipulation of the culture environment during differentiation of hNPs altered the percentage of neuronal subtype derivates. Removal of the caudalizing agent RA concurrent with alteration of growth factors involved in brain phenotype specification resulted in differentiation of 5HT^+^/ChAT^+^ cells similar to those of the raphe dorsalis, DARPP32^+^ cells similar to those of the striatum, and TH^+^ cells similar to those of the nigrostriatal pathway. These data suggests that hNPs are an important tool for the discovery of novel targets and factors that influence neural sub-type differentiation. Glial cells remained absent or minimal in the manipulated culture conditions, confirming the restriction of hNPs to the neuronal lineage.

The differentiation of transplanted cells is dictated by their innate differentiation potential, as well as the environmental signals and cellular deficiencies at the site of implantation [24]. Transplantation of hNPs into the injured adult spinal cord resulted in differentiation to neuronal phenotypes. TUJ1 and doublecortin immunolabeling demonstrated that hNPs retained their neuronal identity. Some human cells expressed the p75 neurotrophin receptor indicating young motor, sensory and sympathetic neuronal phenotypes, while others expressed GAD 65–67 and ChAT indicating interneurons and cholinergic neurons respectively [25]. The injured spinal cord is thought to be innately gliogenic, as transplantation of multipotent neural stem cells [26] or spinal cord progenitor cells [27], [28], [29] results in glial differentiation, even when they are primed to become cholinergic neurons [30]. Thus, these data indicate that transplanted hNPs retain their neuronal differentiation potential within a gliogenic environment.

These data demonstrate the production of hNPs in large quantity and high purity from hESCs in serum-free and feeder-free conditions. This cell population is ideally suited for neuronal screening and neuronal developmental studies, as well as therapeutic strategies addressing diseases and conditions characterized by the loss of neurons. Importantly, this cell population represents a starting point for the directed differentiation of neuronal sub-types.
